# Impact of Age on Patients’ Communication and Technology Preferences in the Era of Meaningful Use: Mixed Methods Study

**DOI:** 10.2196/13470

**Published:** 2020-06-01

**Authors:** Martina A Clarke, Ann L Fruhling, Marilyn Sitorius, Thomas A Windle, Tamara L Bernard, John R Windle

**Affiliations:** 1 Division of Cardiovascular Medicine Department of Internal Medicine University of Nebraska Medical Center Omaha, NE United States; 2 School of Interdisciplinary Informatics College of Information Science and Technology University of Nebraska Omaha Omaha, NE United States; 3 College of Public Health University of Nebraska Medical Center Omaha, NE United States

**Keywords:** patients, personal health record, needs assessment, data display, communication, age groups

## Abstract

**Background:**

Identifying effective means of communication between patients and their health care providers has a positive impact on patients’ satisfaction, adherence, and health-related outcomes.

**Objective:**

This study aimed to identify the impact of patients’ age on their communication and technology preferences when managing their health. We hypothesize that a patient’s age affects their communication and technology preferences when interacting with clinicians and managing their health.

**Methods:**

A mixed methods study was conducted to identify the preferences of patients with cardiovascular diseases. Results were analyzed based on the patients’ age. Grounded theory was used to analyze the qualitative data. Patients were recruited based on age, gender, ethnicity, and zip code.

**Results:**

A total of 104 patients were recruited: 34 young adults (19-39 years), 33 middle aged (40-64), and 37 senior citizens (>65). Young adults (mean 8.29, SD 1.66) reported higher computer self-efficacy than middle-aged participants (mean 5.56, SD 3.43; *P*<.05) and senior citizens (mean 47.55, SD 31.23; *P*<.05). Qualitative analysis identified the following three themes: (1) patient engagement (young adults favored mobile technologies and text messaging, middle-aged patients preferred phone calls, and senior citizens preferred direct interactions with the health care provider); (2) patient safety (young adults preferred electronic after-visit summaries [AVS] and medication reconciliation over the internet; middle-aged patients preferred paper-based or emailed AVS and medication reconciliation in person; senior citizens preferred paper-based summaries and in-person medication reconciliation); (3) technology (young adults preferred smartphones and middle-aged patients and senior citizens preferred tablets or PCs). Middle-aged patients were more concerned about computer security than any other group. A unique finding among senior citizens was the desire for caregivers to have access to their personal health record (PHR).

**Conclusions:**

Patients of different ages have different communication and technology preferences and different preferences with respect to how they would like information presented to them and how they wish to interact with their provider. The PHR is one approach to improving patient engagement, but nontechnological options need to be sustained to support all patients.

## Introduction

### Background

The purpose of the Health Information Technology for Economic and Clinical Health Act was to promote the adoption and meaningful use of health information technology (HIT) [1]. Meaningful use is an endeavor initiated by the Centers for Medicare and Medicaid Services (CMS) and the Office of the National Coordinator for Health IT. It involves the use of certified electronic health records (EHR) fundamentally to improve the quality, safety, and efficiency of care [2]. One of the meaningful use requirements is to engage patients and families in their health by offering secure, Web-based access to patients’ health information and provide tools that support electronic communication between patients and providers [3]. A more robust understanding of patient communication and technology preferences is essential for ensuring that patients have access to relevant information needed to make informed health care decisions [4-7]. Providing patients access to personal health information has the potential to enhance the delivery of health care services and encourage patient engagement [8]. Patient experience is an integral component of health care quality and focuses on aspects of health care delivery, such as getting timely appointments, easy information access, and effective communication with health care providers. Accurate perception of the patient experience is an essential step in moving toward patient-centered care [4,9]. Patient-centered care is 1 of the 6 major domains of health care quality and is defined as “providing care that is respectful of and responsive to individual patient preferences, needs, and values and ensuring that patients’ values guide all clinical decisions [10].” Identifying effective means of communication between patients and their health care providers have a positive impact on patients’ satisfaction, adherence, and health-related outcomes [11-13]. This allows for critical and necessary communication to enable the highest level of patient-centered care possible.

Electronic health (eHealth) is an effective medium for bringing HIT to patients in ways that are easily accessible [14]. eHealth is “*health services and informational resources delivered or enhanced through the Internet and related information and communication technologies* [15].” eHealth supports communication between health care providers and their patients and promotes patients’ self-management of chronic diseases [16-19]. As the use of eHealth increases, it is crucial to examine sociodemographic characteristics, such as age, that may affect patients’ ability to use eHealth [20-26]. Younger adults are historically more open to using eHealth than older adults [25,27-35]. Not all older adults own internet-connected devices to access eHealth tools or have the necessary skills needed to use it, increasing the digital divide gap [27,32,33,35].

With increased access to the internet, digital divide research has shifted from internet connectivity barriers (level one of the digital divide) to barriers related to internet skills and usage (level two of the digital divide) [36]. The third level of the digital divide focuses on individuals’ capacity to translate their internet access and use into favorable tangible outcomes [37-39]. The digital divide has the potential to intensify health disparities among vulnerable populations; previous research provides evidence of the digital divide being a barrier to eHealth adoption [24,26,40-49]. Providing effective eHealth interventions is an opportunity to provide patients with the necessary communication tools for self-management [25,50,51]. This opportunity could be easily missed if patients’ needs, preferences, and abilities are neglected [14].

### Objectives

This study’s objective was to identify the impact of patients’ age on their communication and technology preferences when managing their health. Communication and trust are at the core of the patient-clinician interaction. In addition, technology adoption outside of health care is heavily influenced by a user’s age [52]. Thus, we investigated the communication and technology preferences of patients with cardiovascular diseases in the age of CMS’ Promoting Interoperability Program. Dholakia [52] presents a general framework that an individual’s age is a critical determinant of a person's attitude, use, and perceived benefits of many technologies. Age is influential in technology use and adoption because the periods of a person’s life, notably during years as a youth, shape the skills, orientations, and environments of particular technologies [52]. Therefore, we hypothesized that communication and technology preferences are determined, in part, by the age of the patient. The study aimed to test this hypothesis with the following research question: How does age impact patients’ preferences when managing their health?

## Methods

### Study Design

A mixed methods approach was applied to examine communication and technology preferences by conducting interviews with 104 cardiovascular medicine patients at the University of Nebraska Medical Center (UNMC) in the United States of America. UNMC’s Institutional Review Board approved the study.

### Organizational Setting

UNMC is a Midwest academic medical center whose clinical partner is Nebraska Medicine. The Division of Cardiovascular Medicine operates three clinics with over 28,000 annual patient visits. The Health care Information and Management Systems Society, a nonprofit organization that examines hospitals’ advancement toward implementing HIT, has honored UNMC with Stage 7 of the Electronic Medical Record Adoption Model [53]. Stage 7 means UNMC’s hospitals and clinics have a fully integrated EHR, employ Continuity of Care Documents to transport data, use data warehousing to assess clinical data, and demonstrate summary data continuity for all hospital services [54]. The personal health record (PHR) offered at UNMC’s is Epic MyChart, a tethered PHR.

### Participants

Patients with heart disease were chosen for this study because cardiovascular disease is a complex, chronic disease and is one of the leading causes of death in the United States. Heart disease and stroke account for over 800,000 deaths annually, costing US $316 billion in health care expenditures and lost productivity yearly [55]. Although patients were recruited from the cardiovascular clinic, patients in this sample had comorbidities. A cardiovascular medicine research nurse coordinator recruited patients after their clinic visit based on age, gender, race, ethnicity, and zip code. Zip codes were used to determine urban versus rural environments and establish average household income. Rural areas were defined as a population less than 10,000 and nonadjacent to a metropolitan area. Income was based on census data of median household income for that zip code. Participants were grouped according to their age. Younger participants were categorized as *young adults* (19-39), senior citizens in the US begin at age 65 and were categorized as *senior citizens* in this study, and middle aged were categorized as adults in between the age ranges of the young adults and senior citizens (40-64). Data collection sessions were scheduled and conducted in a clinic or adjacent conference room. Whenever possible, the research interview was linked to their scheduled appointment. Participants were not compensated for their participation.

### Data Collection

Each data collection session lasted 30 to 60 min. After the consent was obtained, sociodemographic data were obtained, and instruments to measure health literacy, confidence using technology, and patient activation were administered followed by the interview. Health literacy was measured using three health literacy screening questions for detecting patients with inadequate or marginal health literacy using a 5-point Likert scale [56]. Confidence using technology was measured using the Computer Self-Efficacy Survey, which is a 10-item survey using a 10-point Likert scale to measure a person’s ability to use a computer [57]. Patient activation was measured using the Patient Activation Measure (Insignia Health), which is a 13-question survey using a 4-point Likert scale to measure the level of patients’ engagement in their health [58].

Participants were interviewed using a semistructured interview guide as a part of the grounded theory approach. The session intended to have an open dialog with patients to understand how they currently receive their clinical care. The moderator discussed with the participants a typical clinic visit asking exploratory questions about their care, communication preferences, use of technology, and patient portal, use if any. Members of the research team asked relevant follow-up questions based on participants’ responses. Sampling continued until saturation was reached and no new information was gained during interviews. Study timeframe was from July 2015 to November 2018.

### Data Analysis

A mixed methods approach was used to collect and analyze data. Survey data were recorded in a secure database and analyzed in conjunction with a biostatistician using SAS 9.4. Descriptive statistics, which included counts and percentages, means, standard deviations, medians, minimums, and maximums were used to summarize the data. Patient demographics were compared between age categories using the Fisher exact test for categorical data. Comparisons of scores between age groups were done using the Kruskal-Wallis test. Pairwise comparisons were adjusted using Bonferroni method if the overall *P* value from the Kruskal-Wallis test was significant. All tests were two-sided and a *P*<.05 was considered statistically significant.

The audio-recorded interviews were stored on a secure server. Grounded theory approach was used to analyze the qualitative data. Concurrent data collection and analysis were conducted through various stages of coding. Audio files were transcribed, and two independent, qualitative coders analyzed the transcribed interviews. A codebook was created, and themes were revised until coders came to a consensus. A third independent reviewer settled any disagreements between the two coders. Qualitative data were analyzed using NVivo 11, a qualitative data analysis software (QSR International, Doncaster, Australia).

## Results

### Participant Characteristics

Table 1 shows the demographics of patients with cardiovascular diseases who participated in the study and whose ages ranged from 19 to 95 years. Responses are classified according to age groups: young adults: 19-39 years of age (34 participants), middle aged: ages 40-64 (33 participants), and senior citizens’ age >65 (37 participants). A difference was found among race across the three age groups.

There is no significant difference among the three age groups’ health literacy (*P=*.10) and activation level (*P=*.09). There is a significant difference between at least 2 of the 3 age groups in computer self-efficacy (Table 2). Young adults report higher computer self-efficacy than middle-aged participants (*P<*.05) and senior citizens (*P<*.05).

**Table 1:** Demographics of 104 patients with cardiovascular diseases who participated in interviews presented as percentages. Examined demographics include gender, race, age, location, income, and personal health record use.

Demographics			Aged 19-39 years, n (%)		Aged 40-64 years, n (%)		Aged >65 years, n (%)		*P* value
Age (mean 55 years)			34 (33)		33 (32)		37 (36)		
**Gender**								.49	
	Male	14 (41)		19 (56)		18 (53)			
	Female	20 (59)		14 (41)		19 (56)			
**Race and ethnicity**								.02	
	African American	3 (9)		10 (29)		4 (12)			
	Hispanic	6 (18)		1 (3)		1 (3)			
	White	24 (71)		22 (65)		30 (88)			
	Asian	1 (3)		0 (0)		0 (0)			
	American Indian	0 (0)		0 (0)		2 (6)			
**Location by zip codes**								.14	
	Rural (population<10,000)	11 (32)		7 (21)		12 (35)			
	Urban (population>10,000)	23 (68)		26 (76)		25 (74)			
**Income by zip codes**								.69	
	Average household income <US $45,000	5 (15)		12 (35)		10 (29)			
	Average household income >US $45,000	29 (85)		21 (62)		27 (79)			
**Personal health record** **user**								.55	
	Yes	26 (76)		17 (50)		19 (56)			
	No	8 (24)		16 (47)		18 (53)			

**Table 2:** Comparison of composite scores of surveys between age groups.

Survey		Count, n	Mean (SD)	Median (min-max)		*P* value^a^
**Computer self-efficacy**					<.001	
	Young adult	34	8.29 (1.66)	9 (3.6-10)		
	Middle aged	30	5.56 (3.43)	5.85 (0-10)		
	Senior	31	4.78 (3.13)	4.6 (0-9.9)		
**Patient activation measure**					.09	
	Young adult	34	72.18 (14.43)	71.34 (51-100)		
	Middle aged	30	63.4 (15.7)	59.38 (44-100)		
	Senior	31	65.24 (20.13)	65.47 (44-100)		
**Health literacy**					.16	
	Young adult	34	2.6 (0.42)	2.33 (2-3.7)		
	Middle aged	30	2.84 (0.5)	2.67 (2-4)		
	Senior	32	2.82 (0.74)	2.67 (1.3-5)		

^a^Pairwise comparisons: young vs middle aged, *P*<.05; young vs senior, *P*<.05.

### Impact of Age on Patients’ Preferences When Managing Their Health

In total, three themes emerged by analyzing and combining findings to form overarching concepts: Patient Engagement, Patient Safety, and Technology.

#### Patient Engagement

Patient Engagement is based on the foundations of trust and communication, which are two common and important sub-themes identified by patients across all demographics. Trust and communication in patient-provider relationships can influence patients’ communication preferences [59].

### Similarities Among Age Groups

Patients view care as continuous rather than episodic (ie, a clinic visit) and expect communication to occur both before and after appointments. Calendar reminders are preferred (paper and electronic) with the option to add appointments directly to their calendar.

When she comes to give me my paper, I will go and start putting it in my calendar. I will put it in my calendar on my phone which also pops up in my tablet because it is through google. I will put it on my calendar at work…

Participant 394-082, age 37

Between visits, patients who use a PHR prefer to communicate electronically with their physicians via their PHR or email. PHR nonusers prefer to communicate in person or over the phone. Irrespective of age, trust is a common subtheme and is vital to all age groups with one exception; middle-aged patients are less likely to trust technology than young adults or senior citizens. Irrespective of age, most patients prefer to complete questionnaires before their clinic visit either over the phone or electronically.

I like it when they send them to you before. I have had that done here, and that is nice. It saves me some time because I don’t live in Omaha. I drive like 45 minutes in. So that is nice.

Participant 394-087, age 35

Patients have strong negative feelings about redundant questioning during their clinic visits. All patients understand the importance of knowing their diagnosis, lab results, and medications and prefer their physician’s impressions, in lay terms, rather than a table of results.

Instead of the big words and I have no idea what it is. That would be great.

Participant 394-030, age 67

### Differences Among Age Groups

Differences in preferences are found between groups (Table 3). Young adults have an interest in communicating with other patients with similar conditions via social groups but strongly prefer those sessions to be moderated by professionals. Young adults also request the ability to update information through the PHR and to communicate with providers through text, email, and secure messaging. Of note, social media is not a priority for middle-aged adults. They prefer to contact their physician by phone or email rather than texting. Senior citizens prefer communication with their providers through phone calls, email, and in person and are not interested in participating through social media.

**Table 3:** Preferences between age groups (young adults, middle-aged adults, and senior citizens) regarding the theme of patient engagement.

Patient engagement^a^	Differences in preferences between age groups
Phone reminder issues	Young adults: Ineffective because they rarely check their voicemail. *Sometimes I get a voicemail, and sometimes I don’t check my voicemail until a week after. I’ve never missed an appointment, but just that I read my texts all the time, so that would be nice.* [Participant 394-017, age 24] Middle-aged adults: Did not mention issues with phone reminders. Messages begin too abruptly, so they miss the beginning. *We older people can't just run and jump to the phone. By the time I pick up the phone, the message is done, and I heard it say 3 o'clock. I heard the 13th. I think that the message that they leave on the phone should wait until somebody picks it up.* [Participant 394-036, age 64] Senior citizens: Miss reminders because of low ringtones. *All I have to do is answer. My problem is that a lot of times I don't hear because the ring isn't so loud, and by the time I get to it, the caller has already hung up.* [Participant 394-018, age 84]
Provider communication	Young adults: Text, secure messaging, email Middle-aged adults: Phone, email Senior citizens: In-person, phone, email
Scheduling appointments	Young adults: In-person after a visit, phone, PHR^b^ Middle-aged adults: Phone, open to using PHR Senior citizens: Phone
Appointment reminders	Young adults: Electronic calendar, text messages Middle-aged adults: Electronic and paper calendar, email Senior citizens: Phone calls, handwritten notes
Repetition	Young adults: Dislike repetitive questions during current check-in Middle-aged adults: Do not mind repetitive questions during check-in Senior citizens: Approve of repetitive questions during the current check-in process because they believe it confirms that the right patient is being treated. *I don't mind. Sometimes you will see five or six different people, and they will all ask the same thing. I think that's good. That way if they have to give you something, they are not giving it to the wrong person.* [Participant 394-002, age 80]
Caregivers	Young adults: Did not mention caregiver access. Middle-aged adults: Some do not want to share information with children because they want to be in control of their health care. Senior citizens: Wants caregivers to have Web access to health information. *M: I have a list of medications I carry with me all the time. She comes to the appointments, and she is my secretary.* *F[spouse]: That way, I know what is going on. I ask, are there any surprises?* [Participant 394-011, age 73]
Lab results	Young adults: Did not mention a preference Middle-aged adults: Phone call, letter Senior citizens: Letters, email
Social engagement	Young adults: Had an interest in social groups with patients with similar diseases. Middle-aged adults and senior citizens: Did not mention an interest in social engagement.

^a^The three age groups categorize the findings. Text in *italics* represents participants’ quotes.

^b^PHR: personal health record.

#### Patient Safety

The quality of health care can be improved by focusing on improving patient safety and is an excellent standard of measure for high-quality health care. Patient safety is the effective prevention and amelioration of the risk of medical-error related adverse events [60].

### Similarities Among Age Groups

Medication reconciliation is critical in ensuring proper medication adherence and avoiding patient harm from inaccurate information [61,62]. All patient groups are frustrated that their home medications often are not reflected in the EHR despite attempts at reconciliation.

Mine [home medication list] is correct, but for some reason, whenever I go in, they have some that are like from 12 years ago. I don’t understand why the doctor’s list is so wrong compared to mine.

Participant 394-017, age 24

The after-visit summary (AVS) reflects a summary of activities that occurred during the clinic visit including lab results, medications and changes, and treatment plans, as well as patient education. All age groups focus on and prefer changes in treatment plans and medications to be highlighted in their AVS.

Age impacts internet use for health information. Patients commonly identify the Google search engine as their health information source. Patients enter their search and select a link from the populated list. They can occasionally name the websites they access for health information. They also request links to credible sources about their diseases.

No, I just kinda Google it and go through anything I can get into. I do not belong to any medical thing. Doctors have told us that is probably not a good idea to do all that but I always do.

Participant 394-011, age 73

Age also impacts travel with health information. Participants carry some part of their health information with them while traveling. They are also open to alternative ways of travelling with their health information when out of town in case of an emergency, such as, on their phones or on a card the size of a debit card.

Not like I should. I mean, I have the thing on my iPhone that is the emergency alert. I put my conditions on that. I guess I kind of, maybe, but it does not have like a list of your medicines or things like that.

Participant 394-084, age 35

…I got a cut deep in my ear from something, and it bled like crazy forever, all of the ways home. It happened in Hawaii, and all of the ways home, we were changing cotton soaked and I came in, and they put me with the ENT out at Oakview [UNMC clinic]. When I went to the hospital, of course, they wanted to know about me before they do anything and so I had to fill out a bunch of paperwork. It would be nice to be able just to hand them the flash drive. They could then plug it in their computer and sync it up.

Participant 394-007, age 77

### Differences Among Age Groups

There are differences between preferences for the AVS and medication reconciliation at clinic visits (Table 4). Young adults expressed little interest in the paper version of the AVS, preferring an electronic version. Middle-aged and senior citizens prefer a paper-based AVS.

Young adults and senior citizens are most likely to look up health information on the internet than middle-aged adults. Young adults usually search for information about their condition and the meaning of their lab results. Middle-aged adults search for information about their condition and information on nutrition and weight loss. Senior citizens search for information concerning their medications and the meaning of their lab results.

Young and middle-aged adults report visiting trusted, well-known websites for their health information, citing WebMD and hospital websites, such as Mayo Clinic. Young adults verify the information they receive on the internet by comparing the information provided by multiple sites. Senior citizens determine if the information gathered from the internet is accurate by verifying the information with their physician or pharmacist.

Regarding travelling with health information, some young adults do not want to travel with their health information but are open to doing so in the future when their chronic disease is less controlled. Middle-aged adults and senior citizens travel with their current medication list. Senior citizens also carry other pertinent health information, such as medical device information. Young adults and senior citizens are open to traveling with their health information on their phones. Young and middle-aged adults are less enthusiastic about the option of a health information card because the card is another item they would have to keep track of.

**Table 4:** Preferences between age groups (young adults, middle-aged adults, and senior citizens) regarding the theme of patient safety.

Patient safety	Differences in preferences between age groups
AVS^a^	Young adults: Did not find AVS very useful Preferred an electronic copy Middle-aged adults: Focused on highlighted sections in AVS. Preferred paper copies for record-keeping. Senior citizens: Focused on highlighted sections in AVS. Preferred paper copies for record-keeping.
Medication reconciliation^b^	Young adults: Did not mention issues with the medication reconciliation process Middle-aged adults: Carry medication list to clinic visits. Senior citizens: Prefer to complete before clinic visit electronically or over the phone Carry medication list to clinic visits.
Navigation	Young adults: Did not mention navigational functionality within the hospital. Middle-aged adults: Did not mention navigational functionality within the hospital. Senior citizens: Desire navigational functionality within the hospital, reporting getting lost more than once in hospitals.
Traveling with health information	Young adults: Rarely travel with health information but open to doing so on the smartphones *When I used to go out in Colorado Springs to visit my boyfriend’s family, I would tell his mom everything and give her paperwork. So, that way then, in case something happens to me for all I know, I’ll get run over by a bull, you know, eaten by a mountain lion.* [Participant 394-089, age 36] Middle-aged adults: Travel with their current medication list Senior citizens: Travel with their current medication list and medical device information
Health information source	Young adults: Trusts well-known health websites (eg, WebMD) and hospital websites (eg, Mayo Clinic) for their health information Middle-aged adults: Trusts well-known health websites (eg, WebMD) and hospital websites (eg, Mayo Clinic) for their health information Senior citizens: Identifies mainly Google as their health information source *No, I am just on Google and I just type in the medicine’s name and look it up and see what the side effects are and stuff like that. They show pictures of what they used to look like, the different brands, and get information on them.* [Participant 394-046, age 72]
Information search topics	Young adults: Search for information about their condition and the meaning of their lab results *Vagal tone, yes! So like that word, just, I have got to remember all those things, so it’s nice to be able to go on there and you know, read things like that.* [Participant 394-034, age 28] Middle-aged adults: Search for information about their condition and information on nutrition and weight loss. Senior citizens: Search for information concerning their medications and the meaning of their lab results.

^a^AVS: after-visit summaries.

**^b^**The 3 age groups categorize the findings. Text in *italics* represents participants’ quotes.

#### Technology

The technology theme focuses on attitudes, device preferences, and password management.

### Similarities Among Age Groups

All age groups are open to using a swipe card similar in size to a debit card for checking into their clinic visit. Participants in all age groups find the tables and graphs in the PHR hard to understand. They also prefer a phone call when receiving abnormal test results to allow a dialog concerning the results.

…when sometimes things could be abnormal and need to be discussed, the phone call would be better.

Participant 394-003, age 65

They also desire medication information in the PHR.

What I don't find useful, when he takes his meds, it usually just has your dosage you take and what it is, the name of it, but it doesn't tell you, and I think it should tell you, because he takes so many meds, why, what is this for. Like today, this is for your cholesterol. That is why you are taking it. Because for him, when he goes from doctor to doctor, he has so many, he forgets, and I'll even forget because he just takes so much, so I do think it should say something like that.

[Participant 394-037, age 57

Patients find passwords cumbersome to recall and are open to alternative authentication methods for accessing their PHR. Participants attempt to recall their passwords from memory, but to assist with recall, they keep a notebook with a list of their passwords, or they use the same passwords for all the sites they access. They also confess to sharing their passwords with others.

I have basically one password for everything I do, and then every six months, I change it…

Participant 394-001, age 57

I have a book of passwords at home that if she [spouse] needed to get to it, she could get to it.

Participant 394-004, age 67

All age groups desire the ability to have electronic access to laboratory and test results with normal values and their trends in the PHR.

The synopsis from the doctor looking at it would be my guess. The option of looking at it just for curiosity, but that wouldn’t make as much difference as what the doctor would have to say because unless I’m trained in the field, I wouldn’t know what I was looking at anyway.

Participant 394-013, age 65

### Differences Among Age Groups

Young adults prefer to access their PHR through an app on their smartphones (Table 5). Young adults are willing to complete the check-in process for their clinic visits using their phones and are open to Web-based audiovisual clinic visits. Middle-aged participants prefer a PC or tablet to smartphones. Senior citizens are similar; they prefer a PC or tablet because of the need for a larger font. Middle-aged participants express difficulties navigating the PHR. A unique feature request by senior citizens is the ability to delegate PHR access to caregivers.

Young adults maintain their passwords from memory for fear of misplacing a written list. To mitigate this fear, young adults write their passwords in an encrypted format or keep a list in a notes app on their phone or a computer file. Senior citizens’ main frustration with passwords is that they cannot remember them because of the differing password criteria that websites require. Some senior citizens keep their password list in a computer file. Misplacing paper versions of their password list is rarely a concern and even have passwords written on Post-It notes taped to their desks at home.

**Table 5:** Preferences between age groups (young adults, middle-aged adults, and senior citizens) regarding the theme technology.

Technology^a^	Differences in preferences between age groups
Electronic check-in	Young adult: Very receptive to complete the check-in process for their clinic visit using their phone. Middle-aged adults: Not open to complete the check-in process for their clinic visit using their phone. Senior citizens: Preferred human interaction but were open to complete the check-in process for their clinic visit using their phone. *I would not do FaceTime. I prefer human to human contact.* [Participant 394-015, age 86] *I do not think it would be necessary. It might add to it for some people. I think there are some people that work better in that kind of environment, but that is not necessary for me.* [Participant 394-007, age 77]
Web-based clinic visit	Young adult: Very receptive to the convenience of Web-based audiovisual clinic visits. *If I were master of the universe, I would have FaceTime appointments. I would have it where I can get the lab done, you know, at my local lab link at the local hospital.* [Participant 394-020, age 35] Middle-aged adults: Split when it came to Web-based and audiovisual clinic visit option. Senior citizens: Not receptive to the convenience of Web-based audiovisual clinic visits.
Preference: PHR^b^ access device	Young adult: Smartphone Middle-aged adults: Tablet, computer Senior citizens: Tablet, computer
Preference: internet access device	Young adult: Smartphone Middle-aged adults: Tablet, computer Senior citizens: Tablet, computer
Accessibility	Young adult: No issues Middle-aged adults: Larger font Senior citizens: Larger font
Password management	Young adult: Use the same password, alternate between a few passwords, keep a computer file, Post-it Middle-aged adults: Notebook, use, the same password, spouse Senior citizens: Use the same password, notebook, Post-it, saved in the browser, computer file.
Message received receipt	Young adult: No issues Middle-aged adults: Do not electronically communicate because they don't know if their provider receives messages Senior citizens: Confirmation receipt to know that their message is received

^a^The three age groups categorize the findings. Text in *italics* represents participants’ quotes.

^b^PHR: personal health record.

## Discussion

### Summary of Findings

This study demonstrates the importance of the role that age plays in determining communication preferences and technology use, with age having a significant impact on patient preferences in clinical care. There was a difference between race across the three age groups. This difference was because our sample was representative of the population studied. The US Census Bureau estimates Nebraska’s population to be 78.6% (1,520,445/1,934,408) White, 11.2% (216,654/1,934,408) Hispanic, 5.1% (98,655/1,934,408) Black or African American, 2.6% (52,229/1,934,408) Asian, and 1.4% (29,016/1,934,408) American Indian.

### Principal Findings

Young adults had the highest self-efficacy score among the three age groups. Young adults are most receptive to completing the check-in process for their clinic visit using their phone and would opt for the convenience of Web-based audiovisual clinic visits, prefer smartphone access, and have a high use of the PHRs for convenience. Middle-aged adults had lower computer self-efficacy scores than young adults. Middle-aged adults are blenders of old and newer technologies. They prefer phone and email for provider communication and both electronic as well as paper calendars for appointment reminders. Similar to middle-aged adults, senior citizens had lower computer self-efficacy scores than young adults. Senior citizens in this study prefer phone communication and mention caregivers gaining access to their health information.

### Comparison With Prior Work

Young adults grew up in the age when mobile devices exploded onto the market and are very familiar with these devices. Results from a 2015 survey conducted by Pew Research Center, determining technology device ownership, found that 86% of young adults (aged 18-29 years), 83% percent of adults (aged 30-49 years), 58% percent of middle-aged adults, and 30% of senior citizens owned a smartphone [63]. Similarly, a study by Zallman et al [64] found that being 40 years old and under was associated with a 50% to 90% increase in the odds of preferring text messaging over other modes of communication. Based on Dholakia’s framework [52], this explains young adults’ preference for mobile PHR access, text message, and electronic access to appointment reminders and their AVS. With a higher reported computer self-efficacy, future research should analyze the effects of a user-friendly mobile PHR interface on increasing adoption among young adults.

Middle-aged and senior patients’ tablet use can be explained by the simplified, less intimidating interface of tablets. A study by Jayroe and Wolfram [65] found that older adults had an overall positive experience when using a tablet to complete search tasks. Previous literature is rich with information on young adults and senior citizens’ as it relates to technology adoption. However, there is a lack of information on how to best present information and increase PHR adoption for middle-aged adults. Middle-aged adults and senior citizens share multiple similarities. Middle-aged adults and senior citizens may be content with their current modes of communication technology use and may be reluctant to attempt to keep up with the fast-changing technology environment.

Although senior citizens are usually behind when it concerns technology adoption, Pew’s report on Americans’ Internet Access found that senior citizens had the highest rate of change from 2000 to 2015, with more than half (58%) of all adults aged 65 years and older using the internet [66]. In addition, in a study by Gordon and Thornbrook [51], over 75% of senior citizens reported having access to a desktop or laptop and 25% owned a tablet. These results suggest a narrowing of the digital divide; therefore, it is crucial to support the senior citizens, who are open to eHealth, to adopt digital tools that enable better health care self-management. Senior citizens are also more likely to use technology that has been around longer and that are most familiar. A study by Olsen et al [67], surveying age-related differences in overall usage of technologies, found that older adults were more likely to use technologies, such as telephones, answering machines, credit cards, etc [67]. Senior citizens in this study also prefer phone communication for the sake of human interaction. Olsen et al [67] similarly discovered that older adults frequently made phone calls when the phone was an option. These results suggest that a combination of new and old technology is still imperative to satisfy the communication needs and preferences of all patients. Senior citizens are also more likely to be more concerned about their health and, maybe, feeling vulnerable because of their dependence on informal, family caregivers for their health management. This may be one motivation to providing caregivers with access to their patients’ health information [68-70]. Allowing caregivers improved access to manage senior patient’s health information is a useful feature to build into PHR redesign.

Patients search for health information on the internet using search engines without considering the links they are selecting from the search list. This is a patient safety issue because patients may be consuming misleading information. Providing patients with trusted links to websites and educating patients on how to determine if an information source is credible are two ways to reduce the potential of patient harm from misinformation.

### Limitations

While this study recruited patients based on age, gender, race, ethnicity, and zip code, a continuing study is necessary to confirm, extend, and refine these results, as there may be other complex reasons that may affect results. The sample consists of patients from one health care system, using one patient portal, with similar chronic diseases. Our results need to be confirmed by a larger multisite study. However, patients receive care at institutions outside of Nebraska Medicine and have experience using PHRs from other manufactures. The purpose of this study is not to predict trends for the future, but to determine patients’ current practice, which can inform providers on the information patients seek and can improve the communication providers have with their patients during clinic visits. In addition, these results can inform vendors of patients’ desired functionality for the design and implementation of PHRs to improve the usability of PHRs.

### Conclusions

This study demonstrates that as implemented, mandatory PHR use could increase the digital divide among vulnerable populations. Many patients, especially the elderly and those with low reported computer self-efficacy, find technology a barrier to use. PHR vendors should consider that young adults have a high affinity for electronics. Middle-aged adults are mixers of old and new technologies, and in our sample, many do not trust technology, preferring to interact with humans than with technology. Senior citizens also prefer human interaction but are willing to use technology that is familiar to them. The PHR does not suitably support the communication needs of elderly patients and their caregivers. Patient-centered care needs to support individual patient preferences, which includes nontechnology options.

### Clinical Relevance Statement

To achieve substantial, meaningful use, HIT must adapt to the user, rather than forcing the user to adapt to the technology. Patients of different ages have different communication and technology preferences, information display differences, and preferences regarding how they wish to interact with their provider. The PHR does not correctly support caregiver access to health information. It is one approach to improving patient engagement, but to support all patients, sustaining nontechnological options are necessary.

## Abbreviations

AVS: after-visit summaries
CMS: Centers for Medicare and Medicaid Services
eHealth: electronic health
EHR: electronic health record
HIT: health information technology
PHR: personal health record
UNMC: University of Nebraska Medical Center
